# Serendipitous high-resolution structure of *Escherichia coli* carbonic anhydrase 2

**DOI:** 10.1107/S2053230X25000068

**Published:** 2025-01-15

**Authors:** Michael R. Rankin, Janet L. Smith

**Affiliations:** a Department of Biological Chemistry University of Michigan Ann Arbor MI48109 USA; b Life Sciences Institute University of Michigan Ann Arbor MI48109 USA; The Scripps Research Institute, USA

**Keywords:** contaminants, carbonic anhydrase, *Escherichia coli*

## Abstract

X-ray crystallography remains the dominant method of determining the three-dimensional structure of proteins. Nevertheless, this resource-intensive process may be hindered by the unintended crystallization of contaminant proteins from the expression source. Here, the serendipitous discovery of two novel crystal forms and one new, high-resolution structure of carbonic anhydrase 2 (CA2) from *Escherichia coli* that arose during a crystallization campaign for an un­related target is reported. By comparing unit-cell parameters with those in the PDB, contaminants such as CA2 can be identified, preventing futile molecular-replacement attempts. Crystallographers can use these new lattice parameters to diagnose CA2 contamination in similar experiments.

## Abstract 2

Contaminating proteins from the expression source may inadvertently crystallize instead of the target protein of interest. Two novel crystal forms of *E. coli* carbonic anhydrase 2, as well as a high-resolution (1.43 Å) structure of the enzyme, are reported.

## 1. Introduction

The process of obtaining a three-dimensional structure of a target protein can be time-consuming and expensive, regardless of the technique used. Each stage of the gene-to-structure pipeline has potential for failure, yet the most frustrating and expensive errors may arise at the very end during the analysis of diffraction data. Efforts to solve the structure through molecular replacement or experimental phasing may result in the unfortunate discovery that the crystallized protein was not the target protein.

The PDB contains many accidental structures of contaminants that arose during purification (Niedzialkowska *et al.*, 2016 ▸; Grzechowiak *et al.*, 2021 ▸). Typical purification schemes involve the addition of exogenous proteins such as lysozyme (Falgenhauer *et al.*, 2021 ▸), Tobacco etch virus (TEV) protease (Tropea *et al.*, 2009 ▸) or deoxyribonuclease (DNase) I (Funakoshi *et al.*, 1980 ▸). Any of these proteins has the potential to persist through the purification and crystallize in lieu of the target protein. Genetically encoded fusion proteins such as maltose-binding protein (MBP; Lebendiker & Danieli, 2017 ▸) or glutathione *S*-transferase (GST; Harper & Speicher, 2011 ▸) may also remain in small quantities after cleavage and counterselection.

More commonly, contaminating proteins from the expression source lead to unintended structures. The nickel resin used in immobilized metal-affinity chromatography (IMAC), the most common method used to obtain large quantities of recombinant protein, has the potential to bind proteins other than the polyhistidine-tagged target. Many such contaminants from a nickel-affinity-based *Escherichia coli* purification strategy have been reported (Niedzialkowska *et al.*, 2016 ▸; Grzechowiak *et al.*, 2021 ▸; Bolanos-Garcia & Davies, 2006 ▸). These proteins may bind nickel resin or interact nonspecifically with the protein of interest and thus be retained through the final purification step. Common endogenous *E. coli* contaminants that have been reported to co-elute during nickel-affinity purification include ArnA (Andersen *et al.*, 2013 ▸; Robichon *et al.*, 2011 ▸), SlyD (Andersen *et al.*, 2013 ▸; Robichon *et al.*, 2011 ▸; Parsy *et al.*, 2007 ▸), Hsp60 (GroEL; Bolanos-Garcia & Davies, 2006 ▸), YodA (David *et al.*, 2003 ▸) and Can/YadF (carbonic anhydrase; Chai *et al.*, 2021 ▸). Frequent contaminants are listed in the ContaBase database (Hungler *et al.*, 2016 ▸).

An endogenous carbonic anhydrase frequently contaminates recombinant proteins from *E. coli* expression systems (Robichon *et al.*, 2011 ▸; Chai *et al.*, 2021 ▸; Cronk *et al.*, 2001 ▸; Merlin *et al.*, 2003 ▸). Carbonic anhydrase (EC 4.2.1.1) is a zinc-dependent metalloenzyme that forms carbonic acid from CO_2_, a byproduct of carbohydrate and fat catabolism. In humans, carbonic anhydrases in red blood cells reversibly solubilize CO_2_ as carbonic acid, allowing it to reach the lungs to be exhaled (Doyle & Cooper, 2024 ▸). *E. coli* contains two carbonic anhydrase genes. The essential *can* gene (previously *yadF*; UniProt P61517) encodes carbonic anhydrase 2 (CA2), a β-class CA enzyme. CynT (UniProt P0ABE9) is a paralog of CA2 (33% sequence identity) that can complement disruption of *can* (Merlin *et al.*, 2003 ▸). The PDB contains several structures of *E. coli* CA2, but none of CynT (Table 1 ▸).

Here, we report a case of persistent CA2 contamination that crystallized in three forms. Two are new crystal forms and the third yielded a high-resolution (1.43 Å) structure of a common CA2 crystal form.

## 2. Materials and methods

### 2.1. Protein production

The gene encoding a natural product biosynthetic protein of interest was cloned into the vector pMCSG7 using a ligation-independent cloning strategy (Stols *et al.*, 2002 ▸). To facilitate phosphopantetheinylation of the target protein, the plasmid was transformed into the *E. coli* BL21(DE3) BAP1 cell line (Pfeifer *et al.*, 2001 ▸), which constitutively expresses *sfp*, encoding a nonspecific phosphopantetheinyl transferase (Quadri *et al.*, 1998 ▸). The expression strain also contained the pRare2-CDF (Whicher *et al.*, 2013 ▸) plasmid. These cells were made competent by the Mix & Go! *E. coli* Transformation Kit (Zymo Research). Terrific Broth (TB) cultures containing 100 µg ml^−1^ ampicillin and 50 µg ml^−1^ spectinomycin were grown at 37°C with shaking at 225 rev min^−1^ until an OD_600_ of 1.0 was reached. The cultures were cooled to 20°C for 1 h, induced with 200 µ*M* isopropyl β-d-1-thiogalactopyranoside (IPTG) and 2 g l^−1^l-arabinose, grown for 18 h and harvested by centrifugation at 12 000*g*.

The cell pellet from a 1 l culture was resuspended in 70 ml lysis buffer [50 m*M* HEPES pH 7.8, 300 m*M* NaCl, 10%(*v*/*v*) glycerol, 20 m*M* imidazole pH 7.8], augmented with 1 mg ml^−1^ chicken lysozyme (Sigma), 50 µg ml^−1^ bovine DNase I (Sigma) and 2 m*M* MgCl_2_, and then incubated for 30 min at room temperature with agitation. Complete lysis was achieved via sonication (Branson Sonifier 450). Following centrifugation at 30 000*g*, the soluble fraction was collected, filtered (0.45 µm Millex-HP PES membrane filter unit, Millipore), incubated for 2 h with 5 ml packed Ni–NTA agarose beads (Qiagen) and loaded onto a glass chromatography column (Bio-Rad). The beads were washed with 100 ml lysis buffer before the protein was eluted in 40 ml elution buffer [50 m*M* HEPES pH 7.8, 300 m*M* NaCl, 10%(*v*/*v*) glycerol, 400 m*M* imidazole pH 7.8].

The eluate was then concentrated to 15 ml using a centrifugal filter unit (Amicon) with a 30 kDa molecular-weight cutoff (MWCO) before being diluted to 50 ml in gel-filtration buffer [50 m*M* HEPES pH 7, 50 m*M* NaCl, 10%(*v*/*v*) glycerol]. The diluted protein solution was passed through a 5 ml HiTrap Q HP anion-exchange column (Cytiva) at a flow rate of 3 ml min^−1^. Proteins were fractionated by a NaCl gradient (50–400 m*M* over 125 ml).

**Figure 1:**
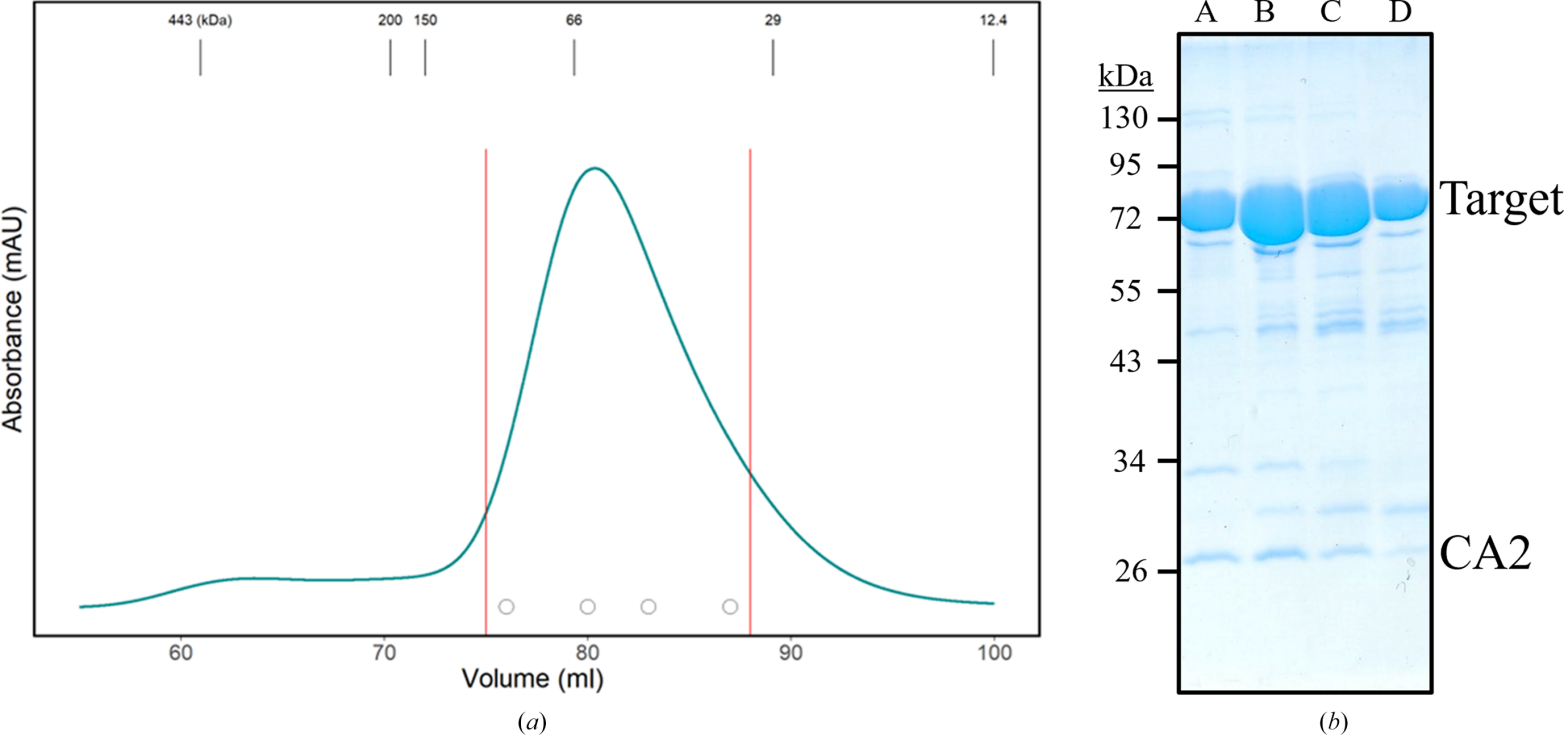
Assessment of protein purification. (*a*) Size-exclusion chromatography indicates that the target protein (72.6 kDa) is monomeric. Masses of molecular-weight standards are indicated at the top. Red lines indicate the pooled fractions, while the four circles correspond to the elution fractions shown in (*b*). (*b*) SDS–PAGE of peak Superdex 200 fractions. Several co-purified contaminants are present, including CA2 (25.0 kDa).

For a final purification step by gel filtration, proteins were concentrated to 5 ml and injected onto a Superdex 200 HiLoad 16/60 prep-grade gel-filtration column (GE Healthcare) that had been pre-equilibrated with gel-filtration buffer. Eluates were assessed by SDS–PAGE (Fig. 1 ▸). The target protein was obtained with an estimated purity of >95% and a CA2 fraction of <1%. Target fractions were pooled, concentrated, flash-frozen in liquid nitrogen and stored at −80°C.

### 2.2. Protein crystallization

The protein sample prepared above was thawed on ice and then dialyzed into a buffer consisting of 10 m*M* HEPES pH 7.8, 25 m*M* NaCl overnight at 4°C using Slide-A-Lyzer MINI dialysis cups with a 10 kDa MWCO. The protein was then concentrated to 8.2 mg ml^−1^ and broad screening with the MCSG suite (Microlytic) was performed using a Gryphon crystallization robot. Within three days, crystals of diverse morphology grew in many conditions (Table 2 ▸). Crystals were harvested directly from the growth conditions and cryoprotected by plunging them into liquid nitrogen. After discovering that these crystals from the initial broad screen did not contain the protein of interest, they were not optimized further. This expression construct was abandoned in favour of a strategy that yielded a sample with higher purity.

### 2.3. Data collection and processing

Data were reduced and scaled using *XDS* (Kabsch, 2010 ▸; Table 3 ▸).

### 2.4. Structure solution and refinement

Molecular replacement (MR) in *Phaser* (McCoy *et al.*, 2007 ▸) using a homolog of the target protein failed. We then searched the PDB for matching lattice parameters and identified CA2 (PDB entry 1i6p; Cronk *et al.*, 2001 ▸) as a match for CA2 crystal form 2. MR via *Phaser* was then carried out using PDB entry 1i6p as a search model. At this point, CA2 contamination was suspected in the other crystals, so the high-resolution structure (PDB entry 9eat) was used as an MR search model for the CA2 samples in crystal forms 4 and 5. Refinement of all models was performed using iterative rounds of *phenix.refine* (Afonine *et al.*, 2012 ▸) and manual model building in *Coot* (Emsley *et al.*, 2010 ▸). The data from crystal form 2 exhibited a strong anomalous signal, presumably due to the tightly bound Zn^2+^ ion, so the *f*′ and *f*′′ contributions of Zn^2+^ were refined for this data set. All structural figures were created using *PyMOL* (Schrödinger). Structure validation was performed with *MolProbity* (Chen *et al.*, 2010 ▸). Refinement statistics are summarized in Table 4 ▸.

## 3. Results and discussion

**Figure 2:**
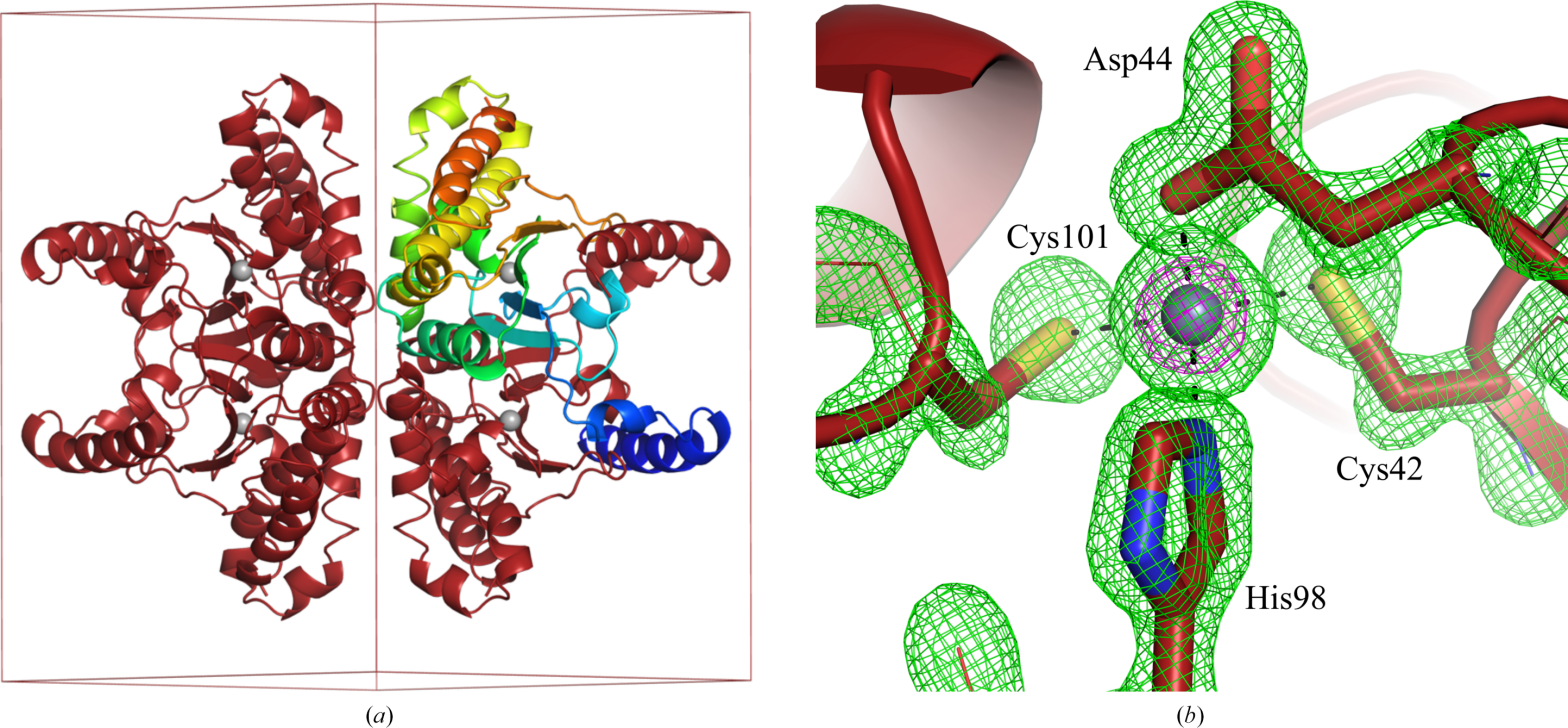
Carbonic anhydrase 2 in crystal form 2. (*a*) The *P*4_2_2_1_2 unit cell contains one subunit per asymmetric unit, with tetramers formed at the 222 centers. The protomer in the asymmetric unit is coloured from the N-terminus (blue) to the C-terminus (red). The bound metal, presumably Zn^2+^, is shown as a grey sphere. (*b*) View of the active site. Polder (Liebschner *et al.*, 2017 ▸) density (green, 7σ) is shown for the presumed Zn^2+^ ion and its coordinating residues. Anomalous difference density is in magenta (20σ).

We report two novel crystal forms of *E. coli* CA2 that was obtained as a purification contaminant. These lattice parameters can be added to the list of crystal forms of CA2, saving time in the case of contamination. In addition to two novel crystal forms, we present a new, high-resolution (1.43 Å) view of CA2 (Fig. 2 ▸). The presumed Zn^2+^ ion is coordinated with tetrahedral geometry by Cys42, Asp44, His98 and Cys101. The coordinate bond lengths, as seen in the high-resolution structure, are Zn—SG(Cys42) at 2.2 Å (range 2.2–2.2 Å), Zn—OD2(Asp44) at 2.1 Å (range 1.9–2.1 Å), Zn—NE2(His98) at 2.1 Å (range 2.0–2.1 Å) and Zn—SG(Cys101) at 2.3 Å (range 2.2–2.3 Å), with the ranges reflecting observations from all models reported in this study.

The identity of a protein in a crystal is technically uncertain until the structure is solved. When structure solution fails despite high-quality data with no obvious pathologies, the contents of the crystal should be considered. This study highlights the unfortunate reality that even off-target macromolecules with low (<1%) abundance may readily crystallize. It is always useful to search the PDB for a unit cell with symmetry and cell constants that match the indexed data.

Sometimes, as was the case for the data sets resulting from CA2 crystal forms 4 and 5, there is no match for the cell and symmetry in the PDB. The fortuitous discovery of a CA2 crystal in the previously characterized form 2 from the same protein sample revealed the contaminant (Table 1 ▸). This observation led to successful MR structure determinations for the other two data sets with CA2 as a search model, yielding CA2 structures in crystal forms 4 and 5.

When working with a new data set that has no matches in the PDB, alternative diagnostic approaches are available. If the amount of crystalline material permits, the size of the crystallized macromolecule may be estimated by SDS–PAGE, or more accurately assessed by mass spectrometry. While researchers have had success with an exhaustive, iterative MR campaign using the full PDB as search models (Keegan *et al.*, 2016 ▸), tools have been developed to solve contaminant structures by using MR more efficiently. *MarathonMR* uses a subset of the PDB based on fold families (Hatti *et al.*, 2017 ▸). For common contaminants, *ContaMiner* performs automated MR against common suspects in the ContaBase database (Hungler *et al.*, 2016 ▸). *SIMBAD* combines several strategies by first searching unit-cell parameters and then screening for common contaminants, before finally performing a brute-force search of a nonredundant subset of the PDB (Simpkin *et al.*, 2018 ▸).

Sometimes the source of the contaminating protein comes not from the expression source, but from contaminating cells. *Serratia proteamaculans* was suspected to have contaminated *Trichoplusia ni*, as the cyanate hydratase CynS co-purified with the target protein and formed well diffracting crystals (Butryn *et al.*, 2015 ▸). Mass spectrometry identified CynS, and MR was successful. The *Serratia* genus appears to be notorious for cell contamination, as different laboratories have reported contamination with *Serratia* CynS (Pederzoli *et al.*, 2020 ▸) and glycerol dehydro­genase (Musille & Ortlund, 2014 ▸) when expressing targets in *E. coli*.

Although the interference of contaminating proteins in a structural biology project is frustrating, it may sometimes lead to exciting results. Trace lysozyme added to cells during lysis formed a heterotrimeric complex that facilitated crystallization of the cortactin–Arg complex (Liu *et al.*, 2012 ▸). Crystallographic analysis of co-purified contaminating proteins has also yielded novel structures. Examples include the yeast nicotinamidase Pnc1p (Hu *et al.*, 2007 ▸), the putative cysteine hydrolase YcaC from *Pseudomonas aeruginosa* (Grøftehauge *et al.*, 2015 ▸) and the *Achromobacter *sp. bacterioferritin Dh1f (Dwivedy *et al.*, 2018 ▸).

Engineering approaches may also minimize the chances of co-eluting proteins when using a nickel-affinity purification strategy. Cell lines such as *E. coli* LOBSTR (low background strain) have been developed by modifying the *arnA* and *slyD* genes of *E. coli* BL21(DE3) such that the encoded proteins exhibit weaker binding to Ni–NTA resin (Andersen *et al.*, 2013 ▸). Similarly, in the engineered NiCo21(DE3) *E. coli* strain, DNA encoding a chitin-binding domain is appended to the 3′ ends of *slyD*, *can* and *arnA*, allowing chitin-resin depletion of the corresponding problematic proteins. In this strain, *glmS* has also been altered to produce a protein that binds nickel resin with lower affinity.

While usually unwelcome, crystals resulting from un­intended targets may yield new results. We present a high-quality carbonic anhydrase 2 structure that may serve as a new standard for structural studies. Additionally, we report two additional CA2 structures in new crystal forms, which may save time when others encounter the same problem.

## Supplementary Material

PDB reference: carbonic anhydrase 2, space group *P*4_1_2_1_2, 9eat

PDB reference: space group *P*2_1_2_1_2, 9eaw

PDB reference: space group *C*222_1_, 9ebz

## Figures and Tables

**Table 1:** Current and new structures of *E. coli* carbonic anhydrase 2 in the PDB

PDB code	Space group	*a*, *b*, *c* (Å)	*d*_min_ (Å)	Form	Reference
1i6o	*P*4_3_22	81.2, 81.2, 162.1	2.20	1	Cronk *et al.* (2001 ▸)
2esf	*P*4_3_22	82.9, 82.9, 162.2	2.25	1	Cronk *et al.* (2006 ▸)
1i6p	*P*4_2_2_1_2	68.5, 68.5, 85.9	2.00	2	Cronk *et al.* (2001 ▸)
4znz	*P*4_2_2_1_2	67.9, 67.9, 84.9	2.70	2	Niedzialkowska *et al.* (2016 ▸)
7sev	*P*4_2_2_1_2	67.5, 67.5, 85.2	2.30	2	Chai *et al.* (2021 ▸)
9eat	*P*4_2_2_1_2	67.5, 67.5, 85.1	1.43	2	This work
1t75	*P*4_3_2_1_2	110.4, 110.4, 162.5	2.50	3	—
9eaw	*P*2_1_2_1_2	78.2, 104.5, 48.3	2.26	4	This work
9ebz	*C*222_1_	113.2, 119.1, 161.0	2.66	5	This work

**Table 2:** Crystallization conditions

	PDB entry 9eat (form 2)	PDB entry 9eaw (form 4)	PDB entry 9ebz (form 5)
Method	Sitting-drop vapour diffusion	Sitting-drop vapour diffusion	Sitting-drop vapour diffusion
Plate type	Intelli-Plate 96-3 LVR	Intelli-Plate 96-3 LVR	Intelli-Plate 96-3 LVR
Temperature (K)	293.15	293.15	293.15
Protein concentration (mg ml^−1^)	8.2	8.2	8.2
Buffer composition of protein solution	10 m*M* HEPES pH 7.8, 25 m*M* NaCl	10 m*M* HEPES pH 7.8, 25 m*M* NaCl	10 m*M* HEPES pH 7.8, 25 m*M* NaCl
Composition of reservoir solution	0.1 *M* HEPES pH 7.5, 0.2 *M* lithium sulfate, 25% PEG 3350	0.2 *M* magnesium acetate, 20% PEG 3350	0.2 *M* ammonium tartrate dibasic, 20% PEG 3350
Volume and ratio of drop	0.75 µl, 2:1	0.75 µl, 2:1	0.5 µl, 1:1
Volume of reservoir (µl)	50	50	50

**Table 3:** Data collection and processing Values in parentheses are for the outer shell.

	PDB entry 9eat (form 2)	PDB entry 9eaw (form 4)	PDB entry 9ebz (form 5)
Diffraction source	23-ID-B, APS	23-ID-D, APS	23-ID-D, APS
Wavelength (Å)	1.0332	1.0332	1.0332
Temperature (K)	100	100	100
Detector	EIGER 16M	PILATUS3 6M	PILATUS3 6M
Crystal-to-detector distance (mm)	200	400	400
Rotation range per image (°)	0.2	0.2	0.2
Total rotation range (°)	180	166	180
Exposure time per image (s)	0.2	0.2	0.2
Space group	*P*4_2_2_1_2	*P*2_1_2_1_2	*C*222_1_
*a*, *b*, *c* (Å)	67.524, 67.524, 85.076	78.233, 104.516, 48.256	113.227, 119.145, 161.01
α, β, γ (°)	90, 90, 90	90, 90, 90	90, 90, 90
Mosaicity (°)	0.094	0.187	0.140
Resolution range (Å)	47.77–1.43	43.82–2.26	47.89–2.66
Total No. of reflections	442908 (23724)	113168 (11180)	205437 (21188)
No. of unique reflections	69116 (6625)	19166 (1873)	31559 (3099)
Completeness (%)	99.48 (95.50)	99.80 (99.9)	99.9 (99.8)
Multiplicity	6.41 (3.58)	5.9 (6.0)	6.5 (6.8)
〈*I*/σ(*I*)〉	13.1 (1.4)	7.6 (2.3)	6.0 (0.9)
*R*_meas_	0.108 (1.106)	0.307 (1.391)	0.222 (2.633)
Inner shell *R*_meas_	0.051	0.120	0.098
CC_1/2_	0.998 (0.415)	0.988 (0.515)	0.994 (0.584)
Overall *B* factor from Wilson plot (Å^2^)	14.8	26.9	63.7

**Table 4:** Structure solution and refinement Values in parentheses are for the outer shell.

	PDB entry 9eat (form 2)	PDB entry 9eaw (form 4)	PDB entry 9ebz (form 5)
Resolution range (Å)	47.75–1.43 (1.48–1.43)	43.82–2.26 (2.32–2.26)	47.89–2.66 (2.73–2.66)
Completeness (%)	99.47 (95.49)	99.80 (99.90)	99.90 (99.80)
σ Cutoff	0	0	0
No. of reflections, working set	65375 (6260)	17239 (1206)	312882 (2077)
No. of reflections, test set	3470 (364)	1916 (134)	2005 (144)
Final *R*_work_	0.157 (0.303)	0.197 (0.279)	0.261 (0.476)
Final *R*_free_	0.168 (0.313)	0.262 (0.339)	0.287 (0.498)
No. of non-H atoms			
Total	1951	3536	6831
Protein	1716	3429	6824
Ion	1	2	4
Water	235	105	3
Protein residues	212	424	844
R.m.s. deviations			
Bond lengths (Å)	0.009	0.008	0.004
Angles (°)	1.04	0.95	0.650
Average *B* factors (Å^2^)			
Overall	20.0	37.3	82.2
Protein	18.8	37.3	82.2
Ion	12.1	28.5	83.6
Water	29.24	36.3	70.4
Ramachandran plot			
Most favoured (%)	98.57	95.51	97.27
Allowed (%)	1.43	4.49	2.73
Outliers (%)	0.00	0.00	0.00
